# Sequential social experiences interact to modulate aggression but not brain gene expression in the honey bee (*Apis mellifera*)

**DOI:** 10.1186/s12983-017-0199-8

**Published:** 2017-03-03

**Authors:** Clare C. Rittschof

**Affiliations:** Department of Entomology, University of Kentucky, S-225 Ag. Science Center North, Lexington, KY 40546 USA

**Keywords:** Behavioral genomics, Personality, Social insects, Plasticity, Aggression, Collective behavior, Timescale

## Abstract

**Background:**

In highly structured societies, individuals behave flexibly and cooperatively in order to achieve a particular group-level outcome. However, even in social species, environmental inputs can have long lasting effects on individual behavior, and variable experiences can even result in consistent individual differences and constrained behavioral flexibility. Despite the fact that such constraints on behavior could have implications for behavioral optimization at the social group level, few studies have explored how social experiences accumulate over time, and the mechanistic basis of these effects. In the current study, I evaluate how sequential social experiences affect individual and group level aggressive phenotypes, and individual brain gene expression, in the highly social honey bee (*Apis mellifera*). To do this, I combine a whole colony chronic predator disturbance treatment with a lab-based manipulation of social group composition.

**Results:**

Compared to the undisturbed control, chronically disturbed individuals show lower aggression levels﻿ overall, but also enhanced behavioral flexibility in the second, lab-based social context. Disturbed bees display aggression levels that decline with increasing numbers of more aggressive, undisturbed group members. However, group level aggressive phenotypes are similar regardless of the behavioral tendencies of the individuals that make up the group, ﻿suggesting a combination of underlying behavioral tendency and negative social feedback influences﻿ the aggressive behaviors displayed, particularly in the case of disturbed individuals﻿. An analysis of brain gene expression showed that aggression related biomarker genes reflect an individual’s disturbance history, but not subsequent social group experience or behavioral outcomes.

**Conclusions:**

In highly social animals with collective behavioral phenotypes, social context may mask underlying variation in individual behavioral tendencies. Moreover, gene expression patterns may reflect behavioral tendency, while behavioral outcomes are further regulated by social cues perceived in real-time.

## Background

For social animals, behavioral phenotypes exist at both the individual and group levels [1–8]. Understanding the mechanistic and social factors that shape phenotypes at these two levels remains a fundamental challenge in social behavior research. In some cases, the behavioral tendency of the most extreme group member, or the average tendencies across group members, are good predictors of group-level behavioral phenotypes [9–11]. In highly structured societies however, individuals continuously modulate their behavior in response to social cues from colony mates, a process that optimizes group level phenotypes depending on environmental conditions and pre-set heritable rules [2, 12–17]. As a result, individual behavior varies across social contexts, a phenomenon known as behavioral flexibility [18–20].

Despite the highly flexible nature of individual behavior in complex societies, some social inputs can have long lasting effects on both behavioral tendencies and behavioral flexibility [18, 21], influencing individual behavioral phenotype in novel social scenarios encountered later in life. One well-known context for this phenomenon is that of early-life social experiences, which can have persistent effects throughout life, and may even be robust to additional social inputs [22]. In the field of developmental plasticity, a growing body of literature attempts to predict how individuals weigh information from their past and current environments to optimize their phenotypes [23]. Empirical studies that assess how social experiences accumulate over time to affect individual behavioral outcome and flexibility are also necessary to interpret behavioral optimization at the group level.

In the honey bee (*Apis mellifera*), worker bees perform aggressive behaviors in the context of nest defense, which is a collective activity that is modulated at the colony level by ecological conditions [24–26]. For individuals, responsiveness to aggression inducing cues and the decision to engage in tasks associated with nest defense are influenced both directly by ecological cues and indirectly by social interactions with nestmates; these interactions occur in a variety of contexts throughout both the pre-adult and adult life stages [25, 27–31]. Despite the large degree of social sensitivity inherent to aggressive behaviors, it is unknown whether or how an individual’s sequential social experiences cumulatively influence aggression levels or behavioral flexibility in defensive social contexts. If social information accumulated over time influences behavioral outcome, there may be constraints to individual and group level behavioral plasticity that prevent an optimal response to given ecological conditions [32]. Moreover, the group-level impacts of these cumulative individual effects are both unknown and difficult to measure due to the fact that honey bees live in large, complex societies composed of about 20,000- 40,000+ individual workers [1, 33]. The first goal of the current study is to evaluate whether social experiences early in adult life influence individual behavioral outcome, flexibility, and group level aggressive response in subsequent social contexts. To do this in the highly social honey bee, I combine small scale field and lab based social manipulations and behavioral assays of aggression.

The cumulative effects of social experiences on behavior may depend on the nature of the underlying mechanisms that entrain previous experiences relative to those that regulate behavioral outcome on a more proximal timescale. In some cases, these mechanisms operate at different levels of biological organization; for example, early-life social experiences may affect brain structure, while subsequent experiences modulate brain biochemistry [34]. In the context of honey bee aggression, genomics studies demonstrate that brain gene expression patterns track socially-induced behavioral variation, not only for stable shifts in aggression, but also for more rapid and transient changes in phenotype that occur on the order of minutes [24, 25, 28]. Moreover, shifts in aggression across very different timescales and contexts are associated with transcriptional variation in overlapping sets of genes [25, 28, 35]. These findings predict that transcriptomic patterns will track behavioral outcome associated with sequential social experiences, for example, resulting in an interaction effect of multiple experiences on gene expression. In addition to the behavioral analyses described above, the second goal of the current study is to evaluate how transcriptomic patterns reflect cumulative social experience and whether they parallel behavioral effects. To do this, I analyze a small set of previously published honey bee aggression biomarker genes.

## Methods

I manipulated early adult social experience by implementing a full colony chronic disturbance paradigm following Rittschof and Robinson [25]. Briefly, I constructed two pairs of small colonies made up of about 4000 one-day-old adult bees collected from 8 to 10 source colonies headed by naturally mated queens. One-day-old bees were combined and then assigned randomly across colonies of each pair, such that a wide array of genotypes of European descent were evenly represented across each pair. I marked each bee on the thorax with paint to precisely control colony size, and then introduced a naturally mated queen to each colony. I provisioned hives with *ad libitum* food, including a partial frame of pollen and a full frame of honey. Colonies were provisioned with food because young bees do not begin to forage in strong numbers until 6–7 days of age. However, colonies were allowed to forage freely throughout the experiment (following [25]). I established the hives in an apiary and commenced the chronic disturbance paradigm [25]: one colony of the pair (selected at random) was left undisturbed as a control, while the other colony was exposed to a combination of artificial alarm pheromone (an aggression-inducing social cue) and physical agitation (opening the colony and lifting and dropping frames in a controlled manner) on a chronic basis (twice a day, once in the morning between 08:00 and 10:00 and once in the afternoon between 13:00 and 15:00) over the course of the first 8 days of adult life. Relative to the control, this treatment results in a highly robust and significant decrease in aggressive behavior measured at the colony level. This effect persists for at least 24 h following the final disturbance [36]. Brain biomarker gene expression patterns for chronically disturbed bees are also consistent with low aggression [25, 36]. A range of behavioral groups, including foragers, soldiers, and bees collected from inside the hive show these brain gene expression effects [25], which persist for 48–72 h following the final disturbance treatment [25, 36]. Thus, this artificial manipulation of the social environment results in stable changes in both behavior and gene expression.

Following this early-adulthood disturbance manipulation, I collected bees for a second, laboratory-based manipulation of social context. This experiment involved combining bees originating from the disturbed and undisturbed colonies together into new social groups, and assaying the aggressive behaviors of these groups. This laboratory-based manipulation was derived from the nestmate recognition assay [37]. In previous work we showed that when kept in small groups over a relatively brief time period (overnight), bees originating from different colonies can discriminate their new groupmates from foreign bees and respond aggressively towards an intruder [36]. Thus, bees kept in a small group in the lab develop a social identity that can be used to investigate how individuals behave under different social conditions.

**Fig. 1:**
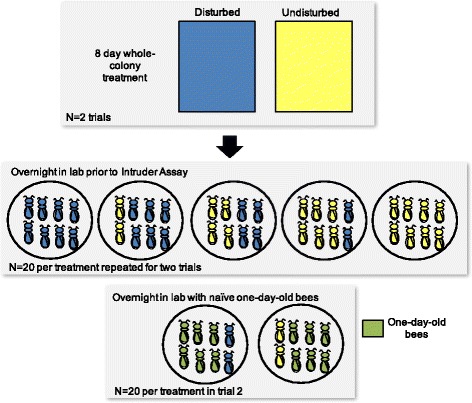
Experimental design. I constructed two pairs of experimental colonies. Each member of the pair was identical in terms of number of individuals, age, and genetic background. One of each pair was disturbed on a chronic basis for 8 days while the other was left undisturbed to generate low (disturbed, *blue*) and high (undisturbed, *yellow*) aggression individuals. We collected and anesthetized individuals, and then combined them into groups of eight for the lab-based assay of aggression. In the second replicate (the second pair of colonies), we added two treatment groups with docile one-day-old adult bees (*green*)

To manipulate the social conditions in the lab, I collected bees from disturbed and undisturbed colonies and combined individuals into small social groups (8 bees per group) that differed in the ratio of disturbed to undisturbed group members, a design analogous to forming groups composed of different numbers of high and low aggression personality individuals ([9], Fig. 1). Groups consisted of 8 undisturbed, 6 undisturbed and 2 disturbed, 4 undisturbed and 4 disturbed, 2 undisturbed and 6 disturbed, or 8 disturbed individuals. I performed these collections on the evening of the 8^th^ day of colony life, 5 h following the final disturbance treatment (prior to collection I first performed a short ~30 s field assay to confirm that disturbed colonies showed the predicted decreased aggressive response compared to undisturbed colonies [25, 36]). To collect enough bees for the groups, I opened each colony and vacuumed bees from the frame containing capped honey. Collecting from the honey frame maximized the chances that I collected roughly the same distribution of bee task groups from both colonies. By 8 days of age, small colonies composed of single-aged bees stratify into a range of behavioral groups (e.g., [38]) including nurses, foragers, and guards. All of these castes could be present on the honey frame and represented in this study. Bees were transferred to plastic bags and anesthetized on ice for ~5 min until sedated. Sedated bees were then transferred into petri dishes in different ratios of disturbed and undisturbed individuals. Sedation is required to eliminate conflict among group members originating from two different colonies (Rittschof, personal observation). I monitored groups until all bees recovered from anesthesia, replacing dead bees as needed. During this monitoring period, I confirmed that there were no aggressive interactions between bees as a result of combining individuals from two different colonies into a single dish. I repeated this entire experiment, including colony construction, chronic disturbance, and the group collections and behavioral across two pairs of colonies. During the second replicate, I added two treatment groups to the 5 listed above. These groups were composed of 6 highly docile one-day-old bees (originating from a single, naturally-mated colony) and either two undisturbed or two disturbed bees. This additional treatment allowed me to investigate how disturbed and undisturbed individuals further alter their behavior in the presence of individuals that are highly docile and largely unresponsive to aggressive cues. In this case, the increased docility is a function of age and not social experience, but both factors contribute to variation in individual aggression in a natural colony context [28], and so it is likely that individual behaviors influence group members in comparable ways. For all groups, bees were provisioned with *ad libitum* with 50% sucrose, and petri dishes were transferred to a dark 34 °C incubator overnight for behavioral assessments beginning the following morning.

Behavioral assessments were performed between 08:00 and 15:00 the following day by two observers. Because bees were paint-marked according to their colony of origin I was unable to blind the experiment. Petri dishes were transferred from the incubator to a temperature-controlled room (25-30 °C) for behavioral analysis. Dishes were arranged in random order and left undisturbed for one hour prior to the initiation of behavioral observations. I assayed individual and group level aggressive behaviors using the Intruder Assay as described in [36]. Briefly, I collected an intruder bee from the entrance of a randomly selected colony, introduced this bee through a small hole into the petri dish containing the eight focal bees, and tallied aggressive behaviors displayed towards the intruder bee. Because all bees were paint-marked, I could assign tallies to either undisturbed or disturbed individuals. Aggressive behaviors include antennation, antennation with mandibles opened, biting, mounting the intruder and flexing the abdomen, and stinging. From these tallies I calculated an aggression index (a tally of aggressive behaviors weighted for severity of behavior, [36, 39]) on a per bee basis, as well as the total level of aggression displayed by the group (on a per bee basis). Following behavioral observations, bees were immediately flash-frozen in liquid nitrogen for later gene expression analysis. Bees were stored separately as a function of social group (the ratio of disturbed to undisturbed bees), but multiple groups of 8 bees were mixed into a single container for storage.

Following protocols described in [25], I used quantitative PCR to evaluate brain expression levels for four biomarker genes. These genes were selected based on previous microarray studies that showed a robust association between brain expression levels and variation in aggressive behavior across social, developmental, and evolutionary contexts [28]. We further validated that these genes are differentially expressed in the brain specifically as a function of chronic disturbance [25]. These four genes are involved in a range of pathways including stress response and alcohol metabolism [25]. Though I have not demonstrated a causal relationship between these genes and aggression level, they are predictive of aggression across many timescales for behavioral variation, and so provide a means to compare the effects of cumulative social experiences on the molecular state of the brain versus behavior.

I dissected brains following Schulz and Robinson [40] and extracted nucleic acids using RNeasy kits including an on-column treatment to remove genomic DNA (Qiagen, Valencia CA, USA). I synthesized cDNA from 200 ng RNA using ArrayScript (Ambion, Life Technologies, Grand Island, NY, USA) reverse transcriptase and a spiked-in internal control to estimate the quality of the synthesis. I performed qPCR on an ABI Prism 7900 in triplicate 10 uL reactions in 384-well plates using PerfeCTa SYBR Green Fastmix (Quanta Biosystems, Gaithersburg, MD, USA). I normalized biomarker genes to the geometric mean of two constitutively expressed control genes, *Actin-1* (*GB44311*) and *Gapdh* (*GB50902*). I verified that control gene expression showed low variance ([41], with Ct standard deviation = 0.18 (*Actin-1*) and 0.20 (*Gapdh*)), and I used two-tailed *t*-tests to verify that expression values did not differ across treatment groups. A stability analysis using GeNorm recommended using the geometric mean of both genes as the endogenous control [42].

All data was analyzed using JMP Pro 12.1. Behavioral data were analyzed using non-parametric statistics because assumptions of normality and equal variance were not met in all comparisons. Observers showed some variation in behavioral scoring, but there were no significant effects of observer on the outcome of any reported results. Gene expression data were analyzed using a relative standard curve method (e.g., [43]), and assessed for normality on a gene by gene basis. All genes except *GB53860* met assumptions for parametric statistical analyses. For *GB53860* I implemented non-parametric tests and generalized linear models (noted in text).

## Results

**Fig. 2:**
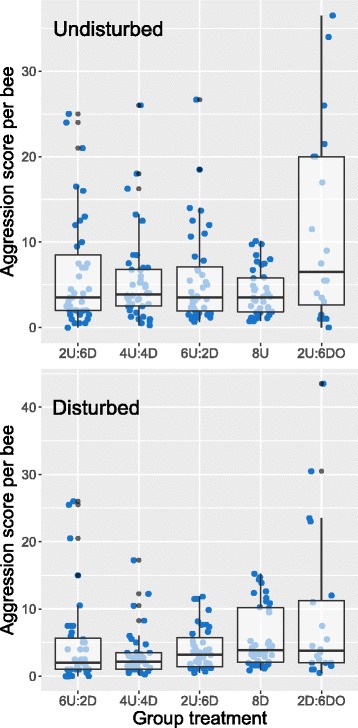
Analysis of aggression as a function of social group. Undisturbed bees showed relatively consistent aggression levels regardless of social group composition while disturbed individuals significantly modulated their aggression in response to social group composition (*top* and *bottom* panels, respectively). “U”, “D”, and “DO” indicate the number of undisturbed, disturbed, and one-day-old bees in each group, respectively. A post-hoc analysis of disturbed bee behavior, using a Wilcoxon Test for each pair, significantly distinguished three treatment categories, 6U:2D and 4U:4D, 2U:6D, and 8D and 2D:6DO. Box hinges show the 1^st^ and 3^rd^ quartiles, whiskers indicate 1.5*IQR from the hinge, and the central tendency line indicates the median. Data points represent scores for individual replicates

**Fig. 3:**
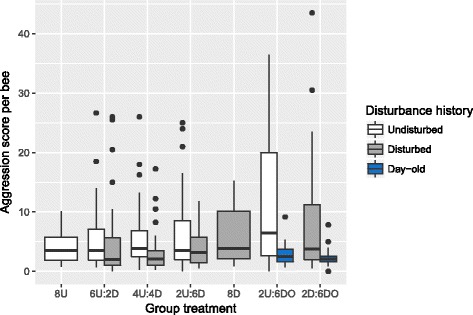
Comparison of aggression scores for undisturbed, disturbed, and one-day-old bees kept in the same social group. A comparison of aggression scores for bees kept together in mixed groups showed that disturbed bees are typically less aggressive than undisturbed bees when kept together (Wilcoxon Exact Test (one-tailed) 6U:2D X^2^_1_ = 4.01, *P* < 0.045, 4U:4D X^2^_1_ = 13.5, *P* < 0.0002, 2U:6D X^1^_2_ = 1.79, *P* < 0.18). Similarly, and as predicted, one-day-old bees were less aggressive than nine-day-old bees, regardless of disturbance history (Wilcoxon Exact Test (one-tailed) 2U:6DO X^2^_1_ = 5.56, *P* < 0.0184, 2D:6DO X^2^_1_ = 4.35, *P* < 0.037). Aggression scores for bees kept in uniform groups (8U, 8D) are shown for comparison. Box hinges show the 1^st^ and 3^rd^ quartiles, whiskers indicate 1.5*IQR from the hinge, and the central tendency line indicates the median

I first compared per-bee aggression scores for each bee type (undisturbed, disturbed, one-day-old) across all small group laboratory aggression assays, regardless of group treatment. As predicted based on previous studies, disturbance history and age significantly predicted aggression scores in this overall analysis (Kruskal-Wallis Test, X^2^_2_ = 12.72, *P* < 0.0017), with undisturbed bees showing the highest average aggression, and one-day-olds showing the lowest. However, a subsequent analysis of aggression score as a function of lab social group and disturbance history showed significant variation in behavior as a function of social group for disturbed bees only (Kruskal-Wallis Test, undisturbed: X^2^_4_ = 4.33, *P* < 0.36, disturbed: X^2^_4_ = 12.86, *P* < 0.012, Fig. 2). Thus, disturbed bees show greater behavioral flexibility as a function of social context compared to undisturbed bees. Disturbed bee aggression increased with decreasing numbers of undisturbed bees in the group, and was highest in groups that contained extremely docile one-day-old bees (Fig. 2). This suggests that disturbed bees increase their aggression levels to compensate for a shortage of high-aggression individuals. Undisturbed individuals show relatively invariant aggression scores as a function of social group treatment, with the exception of groups containing extremely docile one-day-old bees, in which they increase their aggression effort to some degree (Fig. 2). When I compared aggression for disturbed and undisturbed bees kept together in the same social group, disturbed bees were significantly less aggressive in most cases (Wilcoxon Test blocked for group, Fig. 3). As expected, one-day-old bees were significantly less aggressive than their older group counterparts regardless of disturbance experience (Fig. 3).

**Fig. 4:**
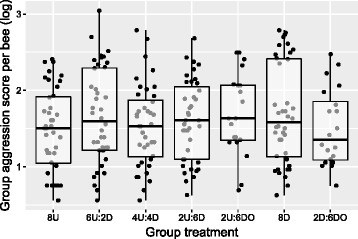
Group aggression score as a function of social group. Total group aggression score did not significantly vary as a function of social group composition. Box hinges show the 1^st^ and 3^rd^ quartiles, whiskers indicate 1.5*IQR from the hinge, and the central tendency line indicates the median. Data points represent scores for individual replicates

Total group aggression scores differed slightly but not significantly as a function of social group composition (Wilcoxon Test, X^2^_6_ = 3.99, *P* = 0.68, Fig. 4). There was no simple relationship between total group score and the prior disturbance experience of individuals within the groups. Notably, the groups that successfully killed the intruder bee had significantly higher aggression scores (Wilcoxon Test, X^2^_1_ = 21.56, *P* < 0.0001), suggesting a strong relationship between scoring methods and a biologically relevant aggression outcome; this outcome, however, also did not vary as a function of group composition (Chi-squared Test, X^2^_6_ = 5.59, *P* = 0.47).

**Fig. 5:**
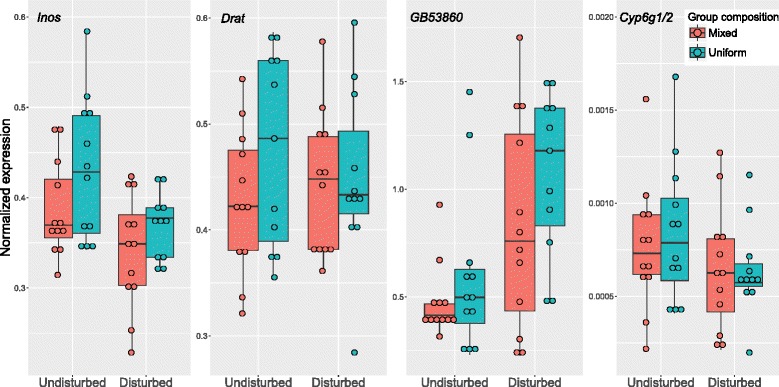
Gene expression data as a function of disturbance history and social group treatment. *Inos*, *GB53860*, and *Cyp6g1/2* showed significant differences in expression in the predicted direction as a function of disturbance history (Table 1, [25]). A pairwise analysis of the effect of social group treatment within each disturbance history treatment showed no significant effects (Table 2), and a series of linear models incorporating disturbance history, social group treatment, and their interaction, showed significant main effects for *Inos*, with no interaction, and no other significant social group treatment or interaction effects for any other genes

To determine whether sequential social experiences influence brain gene expression patterns, I used quantitative PCR to evaluate mRNA levels for four genes identified as aggression biomarkers in a previous study [25]. I evaluated gene expression for disturbed and undisturbed individuals from two group composition treatments, those kept in mixed (4 undisturbed and 4 disturbed) versus uniform (all disturbed or undisturbed) groups. For disturbed bees, individuals across these two social group types showed significant variation in behavior, with higher aggression levels in the uniform groups (Fig. 2). I first assessed whether biomarker expression predicted disturbance history, and found significant effects in the predicted direction for 3 of 4 genes (Table 1, Fig. 5) [25]. However, a subsequent comparison of gene expression level comparing bees kept in mixed versus uniform groups showed that gene expression patterns did not differ as a function of social group treatment even for disturbed individuals (Fig. 5, Table 2). One gene, *Inos*, showed a trend towards significance in the predicted direction for disturbed individuals (*P* < 0.109). A regression analysis for this gene, including disturbance history, lab social group treatment, and their interaction, showed a significant effect of social group across both undisturbed and disturbed individuals, suggesting some effect of social group composition on brain genomic state, but no interaction between the two terms (whole model: F_3,43_ = 4.69, *P* < 0.0064, Disturbance history: F = 9.42, *P* < 0.0037, Lab social group composition: *F* = 4.16, *P* < 0.048, interaction: *F* = 0.224, *P* = 0.64). A significant interaction term would parallel the behavioral results, indicating that chronic disturbance history and social group treatment have synergistic effects on the brain molecular state, similar to their effects on behavior. However, we found no evidence for such a pattern, and little evidence of an effect of social group composition on brain gene expression overall. A similar analysis for the other three genes (but using a generalized linear model with a log link function for *GB53860*) showed no effect of lab social group on gene expression, nor any interaction effects for the sequential social experiences.

**Table 1:** Overall, chronic disturbance had significant effects on brain expression for 3 of 4 aggression biomarker genes

Gene (sample size)	*t/S*	*p*
*Inos* (*N* = 47)	−3.02	**0.002**
*Drat* (*N* = 46)	−2.11	0.417
*GB53860* (*N* = 47)	3.75	**0.0003**
*Cyp6g1/2* (*N* = 47)	−1.70	**0.0483**

Values are the result of one-tailed *t*-tests (or a Wilcoxon Exact Test for *GB53860*) to account for the hypothesized direction of change based on previous studies [25]. Sample sizes represent the total number of individuals compared per test. Significant differences are indicated in bold type face

**Table 2:** Analyzed on a pairwise basis, lab-based social group (mixed, 4U:4D, versus uniform, 8U or 8D) had no effect on gene expression, despite behavioral differences for disturbed bees as a function of group composition (Fig. 2)

	Undisturbed (sample size)	Disturbed (sample size)	Undisturbed	Disturbed
Gene	*t/S*		*p*	
*Inos*	1.61 (*N* = 24)	1.27 (*N* = 23)	0.121	0.109
*Drat*	1.43 (*N* = 23)	0.211 (*N* = 23)	0.166	0.417
*GB53860*	162 (*N* = 24)	154 (*N* = 23)	0.27	0.10
*Cyp6g1/2*	0.54 (*N* = 24)	−0.080 (*N* = 23)	0.598	0.532

*P* values represent the outcomes of two-tailed tests for undisturbed bees (where there was no difference in behavioral expression as a function of social group composition) and one-tailed tests for disturbed bees (following predictions based on the finding that individuals displayed higher aggression levels when kept in uniform groups). Parametric tests were performed for all genes except *GB53860,* which was analyzed using a Wilcoxon Exact Test. Sample sizes represent the total number of individuals compared per test

## Discussion

Results presented here suggest that an individual’s disturbance history and subsequent social context interact to influence aggressive behaviors. Overall, disturbed bees tended to show lower aggression scores relative to their undisturbed counterparts, which suggests that chronic disturbance results in a lower aggression behavioral tendency. However, disturbed bees also showed a high degree of behavioral flexibility, modifying their aggression depending on their social group in the lab-based assay. Conversely, undisturbed bees exhibited similar aggression levels regardless of their social group. As a result of these patterns of behavioral flexibility, lab-based social groups showed similar levels of total aggression, with no clear relationship between group composition and group aggression score. This finding suggests the hypothesis that individual honey bees use negative social feedback to modify their behavior in order to stabilize the group aggression effort. In support of this interpretation, undisturbed bees modulated their aggression effort only when kept in groups composed of highly docile one-day-old bees.

Organized societies are unique in that individuals often modulate their behavior to achieve an optimal group-level phenotype that can be set by ecological conditions or heritable rules [2], which is not always the case for other types of social groups [9]. As a result, individual behavioral outcome is highly sensitive to social context. Honey bee aggression is strongly socially regulated, and the rules individuals use to modulate their behavior seem to depend on the context for nest defense. Previous studies evaluating the effects of social group composition on individual and group level behavior used colony-level manipulations varying ratios of Africanized and European genotypes, which naturally differ in aggression (Africanized bees are more aggressive [29, 31]). When evaluating aggression displayed in the context of mammalian predator defense, studies generally find that individuals adjust their aggression level to that exhibited by the predominant genotype in the nest; European bees express higher aggression levels when kept in a colony with a majority of Africanized individuals, and *vice versa* [28, 31]. As a result, group level defensive effort in the context of a mammalian predator attack is positively correlated with the number of high aggression individuals present in the colony [31]. However, honey bees also defend their nest against smaller arthropod threats, including conspecifics that try to enter the colony and steal honey [44]. In the natural nest context, increased robbing threat from neighboring colonies results in higher numbers of bees guarding the colony entrance at least temporarily [45]. Similar to my present findings, and in contrast to the mammalian defensive response, guarding shows a pattern of social regulation consistent with negative feedback and phenotypic optimization at the group level; European honey bees are less likely to initiate guarding behavior and guard for shorter periods of time when living in colonies composed primarily of the more aggressive Africanized honey bee [29]. Thus, both negative and positive social feedback could play a role in modulating individual aggressive behaviors depending on context. ﻿In a previous study we showed that disturbed colonies have a low overall aggression level in response to a simulated large predator attack [25]. However, in the current study I find that disturbed bees are capable of reaching the levels of aggression exhibited by undisturbed bees. This could be because  disturbed bees are less likely to instigate an attack in a large-predator context, and because  response to large predators is organized by positive instead of negative social feedback [44], the colony mounts a very weak total response. In contrast, in a negative feedback context, disturbed bees are driven to exhibit higher levels of aggression when their group mates are docile. The  lab-based assay, which quantifies response to an intruder bee [37] appears to resemble the guarding context in terms of both the type of aggression stimulus and the apparent rules of social regulation. However, it remains unclear how the target optimum for group aggression level is set and maintained in the field or the lab.

Negative social feedback can enable social species to redirect task effort, e.g., in the context of optimal foraging [46], but more work is needed to understand how the feedback threshold, and thus the group level phenotypic homeostasis, is set﻿ in the context of guarding behavior. I found that group aggression effort was consistent regardless of the sum of individual disturbance histories in a social group. One interpretation of this finding is that a history of chronic predator disturbance does not readily shift the target optimum for group guarding effort. This could be because total group guarding activity is more sensitive to an experience of intruder threat [45], and not the large predator threats simulated by the chronic disturbance (though aggression in these two contexts is correlated to a degree [47]). The optimal guarding effort at the group level is at least somewhat genotype-dependent, as is the social responsiveness of guard bees [29, 47]. 

My results suggest that disturbed bees, which generally show relatively low aggression levels, also show greater behavioral flexibility in response to social group composition compared to undisturbed bees. Studies in species across the sociality spectrum have shown that the degree of behavioral flexibility exhibited by a particular individual can vary as a function of personality. Moreover, in some species, “proactive” individuals, which are often more aggressive, tend to be less responsive to environmental variation compared to low aggression “reactive” individuals [20, 48], consistent with the current results. However, it is difficult to determine whether variation in flexibility is truly an inherent property of disturbed, low-aggression individuals, or rather simply a reflection of the pattern of negative social feedback that regulates guarding behavior [29]. This type of social feedback may cause the appearance of increased behavioral flexibility for low aggression individuals who are less responsive to aggression-inducing social cues and therefore more likely to be socially inhibited during the assay. Very few studies in any species have determine whether individual variation in behavioral flexibility is generalizable or behavioral context dependent [19]. In the honey bee however, there are many established contexts for manipulating the colony environment and evaluating behavioral response [49–51]. Future behavioral studies will address whether patterns of behavioral flexibility are consistent across behavioral contexts or are more easily explained in terms of positive or negative social regulatory paradigms.

I evaluated the effects of sequential social experience on the molecular state of the brain, examining a small set of genes associated with aggression in previous studies [25, 28]. If gene expression patterns reflect sequential social experiences, I predicted a disturbance history by social group interaction on gene expression levels, but no such significant interactions were identified. In general agreement with previous studies, chronic disturbance induced a pattern of brain gene expression consistent with low aggression, but there was little evidence that short-term modulation of social context (i.e., in the lab-based manipulations of small groups) further influenced gene expression. This result stands in contrast to the observed social context-dependent shifts in aggressive behavior for disturbed individuals.

This mismatch between aggression and aggression-relevant brain gene expression, but only in acute social contexts, is intriguing. It could reflect the fact that these selected genes are not causally associated with behavioral change. However, the fact that they are context-dependent predictors of aggression suggests there may be more complex processes involved [52]. For instance, it is possible that these genes are associated with flexibility in aggression rather than aggression level *per se*. However, a previous study [28] found that these same genes predict high aggression in older bees that show a relatively high degree of variation in aggression over time and across individuals (in contrast to ﻿the high aggression undisturbed bees in the current study). Though many studies associate gene expression with behavioral variation, the connection between these two distant phenotypic levels is still poorly understood. There is ample evidence that an ephemeral or acute social experience can induce widespread changes in gene expression [35, 53, 54], but the immediate response to a social situation is likely mediated by neural electrical signaling [55], perhaps even despite existing differences at the molecular level. For example, in migratory locusts, social context instigates a transition from a solitary to a gregarious phenotype associated with swarming [56]. Gene expression changes accompany this transition, but the initial shift in behavior that is required to stimulate changes in other aspects of phenotype (increased or decreased association with conspecifics), occurs rapidly and precedes gene expression changes in some contexts but not others [57]. Similarly, in an ant, the *foraging* gene varies as a function of age but is not a direct predictor of foraging behavior *per se*, despite the fact that foraging activity in general increases with age [58]. Understanding how behavior retains flexibility in some contexts in spite of variation in the molecular or even structural state of the brain presents a challenging area of future work. This work is relevant particularly in light of the fact that an increasing number of studies use gene expression patterns as markers or predictors of consistent individual differences in behavior [59, 60].

In the honey bee, variation in aggression tendency (reflected in gene expression patterns) only becomes obvious if high levels of inhibitory cues are available to disturbed individuals in real time during the behavioral assay. Thus, here I show that a combination of behavioral and gene expression analyses provides an opportunity to identify cryptic variation in personality under conditions in which behavior may appear invariant across individuals. Conversely, these results also emphasize that the existence of consistent individual differences in behavior and behavioral plasticity within individuals are not mutually exclusive alternatives. Finally, gene expression data provide a unique tool not only to explore the neural basis of personality and behavioral flexibility, but also to differentiate transient versus lasting shifts in environmentally responsive behavioral phenotypes [28, 61], particularly in circumstances where personality variation and behavioral flexibility are linked. Here gene expression patterns reflect the more stable behavioral state that results from chronic disturbance, but not behavioral outcomes in real-time due to variation in the relatively short-lived lab based social group context.

## Conclusions

Taken together, the results presented here show that individual behavior is a function of behavioral tendency, social cues experienced in real time, and underlying rules for social modulation of behavior in response to group-level effort. The molecular state of the brain can reveal underlying variation in behavioral tendency and may predict social response, but it does not always match behavioral outcome. In a species with highly socially regulated aggressive behavior, a mechanistic link between aggressive tendency and response to social context provides even greater ability for individuals to fine tune group level behaviors.
